# Plasma Treatment of Cellulose as the First Step in the Synthesis of Second-Generation Biofuel

**DOI:** 10.3390/polym17060782

**Published:** 2025-03-14

**Authors:** Gregor Primc, Miran Mozetič

**Affiliations:** Department of Surface Engineering, Jozef Stefan Institute, Jamova Cesta 39, 1000 Ljubljana, Slovenia; gregor.primc@ijs.si

**Keywords:** biofuel, cellulosic ethanol, depolymerization, plasma

## Abstract

Cellulosic ethanol has been an attractive biofuel for over a century. Despite the large scientific interest, the first step of treating cellulose before enzymatic hydrolysis is still inadequate, so the scientific community seeks innovative solutions. Among them, plasma treatment of raw cellulose represents an interesting approach. The literature on approaches to treat cellulose with gaseous plasma is surveyed, and the results reported by different authors are interpreted. Reactive gaseous particles like ions, electrons, metastables, and radicals interact chemically with the surface but do not cause significant depolymerization of bulk cellulose. Such depolymerization results from bond scission in the bulk cellulose by energetic plasma species capable of penetrating deep into the cellulose. Among them, photons in the range of vacuum ultraviolet radiation (photon energy above the threshold for bond scission) are the most suitable plasma species for the depolymerization of cellulose and the formation of water-soluble fragments, which are suitable for further treatment by enzymatic hydrolysis.

## 1. Introduction

Global warming and a finite stock of fossil fuels stimulate the application of fuels from renewable sources. While “green electricity” is nowadays produced by solar cells, wind turbines, and some other sources, the ethanol for powering vehicles is predominantly synthesized from corn and sugarcane, which are also used as food and feedstock. That is why the broad application of such first-generation biofuels is limited. The ethanol synthesis from cellulose biomass was first reported over a century ago [1] but did not attract much interest from the scientific community until a few decades ago, when the first modern experimental plants were built. The synthesis of cellulosic ethanol involves complex reactions [2,3,4,5,6,7], but basically, it includes four steps: (1) pretreatment, (2) hydrolysis, (3) fermentation, and (4) distillation and dehydration. The pre-treatment should enable at least partial solubility of the lingo-cellulosic biomass, which should facilitate the accessibility of the biomass to enzymatic hydrolysis. This could be achieved by various methods, often employing ionic liquids [8,9,10,11,12] and/or harsh chemicals [13,14,15,16,17,18]. While such a pre-treatment gives encouraging results, it still does not provide the desired depolymerization products using ecologically acceptable methods, which would enable rapid enzymatic hydrolysis and thus decrease the costs [19]. A novel approach to the depolymerization of cellulose materials is treatment with gaseous plasma. Plasma treatment is performed at room or slightly elevated temperatures and does not produce any waste, so it is a promising ecologically benign method for the pre-treatment of lingo-cellulosic biomass. The science behind plasma methods for the depolymerization of cellulose is still in its infancy, and the treatment times are too long for mass application, but it represents an interesting alternative to classical methods. This article reviews the available literature on the plasma depolymerization of cellulose as a pre-treatment step in producing second-generation bioethanol.

Gaseous plasma is often called the fourth state of matter, but in reality, it is a partially ionized gas, i.e., a gas that contains a significant concentration of charged particles (free electrons and positive ions). Gaseous plasma is usually sustained by an electrical discharge, imposed by an electrical field, and the charged particles are accelerated in the field to gain kinetic energy, which is much larger than the kinetic energy of randomly moving gaseous molecules. The ions will share the kinetic energy with neutral molecules, so their temperature in the bulk plasma, far from the electrodes, will be similar to the gas temperature. The electrons lose a marginal fraction of their kinetic energy at elastic collisions with the molecules, so their temperature is significantly higher than the gas temperature. A typical electron temperature in gaseous plasma is between 10,000 and 100,000 K. As with any other randomly moving particles in the gas phase, the electrons assume the Maxwellian energy distribution, so a fraction of electrons will have much higher energy than what corresponds to their temperature. These electrons will cause the ionization, excitation, and/or dissociation of gaseous molecules. As a result, the gaseous plasma sustained in molecular gases will contain a significant amount of molecular fragments, which are chemically extremely reactive and thus capable of chemical modification of organic matter (like cellulose). The electrons also lose their energy due to the excitation of gaseous molecules, i.e., the formation of molecules or atoms in electronically excited states. Such excited particles may return to the ground electronic state by radiation, i.e., the formation of photons. The photon energy corresponds to the energy difference between the excited and the ground state. Many plasmas radiate in the vacuum ultraviolet (VUV) range of wavelengths, corresponding to the photon energy over 6 eV. Such photons are capable of breaking chemical bonds in bulk cellulose because they are not absorbed right on the polymer surface.

## 2. Plasma-Induced Depolymerization

The authors treated both dry cellulose and cellulose suspended in liquids. In the first case, the chemical reactions in bulk cellulose depend on the penetration depth of plasma species in polysaccharides, while in the second case, the reactions are limited by the dissolution of chemically reactive plasma species in the liquid.

### 2.1. Cold, Hot and Thermal Plasmas

As mentioned in the introduction, the energy gained by electrons in an electric field enables a high electron temperature. This is true for all man-made plasmas. The temperature of gaseous molecules, on the other hand, differs from the electron temperature. Plasma is regarded as cold if the gas temperature is close to room temperature. The other extreme occurs when the gas temperature similar to the electron temperature, and such plasmas are regarded as thermal. In between, there are hot plasmas, i.e., the gas is in a non-equilibrium state, but the gas temperature is of the order of 1000 K.

Thermal plasmas are inappropriate for treating solid materials unless melting and/or evaporation is the goal. Namely, gas at the temperature of several 1000 K will significantly heat the surface of any material, including metals with high thermal conductivity. Thermal plasmas are often sustained by powerful discharges, for example, a water–argon DC arc [20]. Cellulose will decompose even after a short treatment with thermal plasma. The treatment of cellulose with hot plasma, sustained for example by gliding arcs [21] or microwave discharges [22], is also not recommended because such plasmas would facilitate the thermal decomposition of cellulose (rather than the depolymerization), which occurs at the temperature of about 540 K [23].

**Figure 1:**
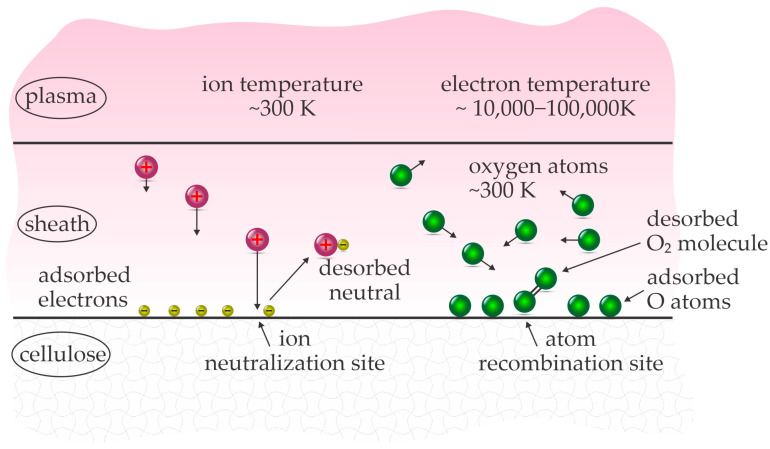
The fluxes of various plasma species and desorption products.

Therefore, only cold plasmas are suitable for treating cellulose in order to depolymerize it. At low pressure, they are typically sustained by radiofrequency discharges [24], and by high-impedance discharges while at atmospheric pressure [25]. In fact, some large industrial low-pressure plasma reactors powered by RF discharges operate at very low discharge power density, often as low as 1 kW/m^3^ [26]. The gas temperature in cold plasma is usually close to the room temperature, and sometimes a bit elevated because of the superelastic gas-phase reactions, say between 300 and 400 K, so still well below the decomposition temperature of cellulose. Regardless of the low gas temperature, the surface of any material exposed to the cold plasma may significantly increase in heat. The energy dissipated on the surface facing the cold plasma will depend on the intensity of exothermic surface reactions. Figure 1 illustrates some exothermic interactions between cold plasma and a solid material.

Heating by the surface neutralization of charged particles, as well as bombardment with positively charged ions from gaseous plasma, is unavoidable. Luckily, the density of charged particles in cold plasma is low, often around 10^15^–10^16^ m^−3^ if plasma is sustained in a continuous mode in a large volume. This range of ion density will provide a flux of ions onto a surface (in the approximation of a plan-parallel geometry) of roughly 10^19^ m^−2^ s^−1^. The sum of ionization and kinetic energy gained across the sheath next to a sample is roughly 30 eV for most gases, so the energy dissipated on the surface of a large object due to the interaction with ions is about 50 W/m^2^, so 0.005 W/cm^2^. This value is a rough approximation, but useful in concluding that the heating by ions is not a limiting factor for the depolymerization of cellulose by plasma treatment.

A sample facing plasma is also heated by the absorption of radiation from cold plasma and relaxation of metastables. The fluxes of metastables of high potential energy and photons are often smaller than the flux of ions, so these two heating mechanisms will not cause the cellulose temperature to be high enough for thermal decomposition. Cold plasmas sustained in noble gases will, therefore, not heat cellulose to such high temperatures.

Noble gases are ideal for sustaining plasmas and are often used by scientists. Practical applications, however, are limited because of the significant cost of noble gases. For economic reasons, plasma is better to be sustained in ambient air, which is actually a mixture of nitrogen, oxygen, carbon dioxide, water vapor, argon, and some other gases and vapors. The rich (and somehow variable) composition of the air makes determining the science behind the interaction between cellulose and air plasma a hard task. One of the most important differences between the noble gas and air plasmas is the richness of endothermic reactions between energetic electrons and air molecules in the gas phase, as well as the richness of the surface exothermic reactions. The exothermic reactions include the heterogeneous surface association of molecular radicals, which cause significant heating of the cellulose surface upon exposure to the cold plasma sustained in the air. The molecular radicals include the recombination of atoms to parent molecules, and the most important one in air plasma is O + O → O_2_. This reaction releases 5.2 eV energy per molecule formed on the surface and may heat materials significantly because of the large density of O atoms in the air plasma.

The above considerations must be taken into account in any attempt to treat cellulose materials with plasma. Namely, the intensity of exothermic surface reactions should be kept at a level that prevents classical thermal degradation. This requirement limits the power (or, better, the power density) used for sustaining plasma for cellulose depolymerization. On the other hand, the exothermic surface reactions will not be harmful to cellulose dispersed in water because they will simply cause more extensive water evaporation.

### 2.2. Treatment of Dry Cellulose Powder

One of the first reports on the depolymerization of cellulose by plasma treatment is the short paper by Benoit et al. [27]. The authors exposed microcrystalline cellulose powder (Avicel PH105) to gaseous plasma sustained in the air at ambient pressure. This type of cellulose is among the most recalcitrant cellulose available on the market. The degree of polymerization of Avicel PH105 is about 200. The cellulose was in the form of dry powder with a particle size below 38 µm. The powder was placed onto one dielectric-barrier-covered electrode of a parallel-plate discharge configuration with a distance between the electrodes of 2 mm. The thickness of the powder film on the electrode was about 0.1 mm. The gaseous discharge was powered at a voltage of 11.2 kV, a frequency of 2 kHz, and the discharge power was estimated to be 26 W. The plasma treatment time was as long as 3 h and resulted in the partial depolymerization of cellulose, since the final degree of polymerization was about 120. The plasma-treated microcrystalline cellulose was mixed with water and A35 catalyst and heated to 150 °C. The authors reported a glucose yield up to 22 wt.%. They also tested glucose production without the catalyst. In this case, the hydrothermal treatment lasted 1 or 3 h, and the glucose yield from plasma-treated cellulose was 5 and 14 wt.%, respectively. The authors also assessed the plasma depolymerization of microcrystalline cellulose of larger particle sizes and found insignificant differences in the glucose yield. The mechanism of plasma depolymerization was not reported, but the authors [27] mentioned the coloring of the cellulose powder, which is probably due to the thermal effects. Unfortunately, the authors did not measure the temperature of the cellulose powder during the plasma treatment.

**Figure 2:**
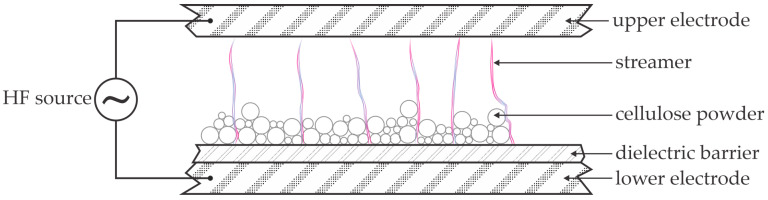
An illustration of the experimental setup used by Benoit et al. [27].

The experimental setup used by Benoit et al. [27] is illustrated in Figure 2. The high-voltage alternating-current (AC) source provides a voltage that would facilitate gas breakdown, according to the Paschen law [28]. The dielectric barrier, however, limits the electrical current, so the discharge between the electrodes is in the form of streamers, i.e., pulses of dense plasma of short duration, typically of a few microseconds [29]. The air in the streamers is partially dissociated and ionized, and gas-phase chemistry facilitates the production of ozone and nitric oxides, whose lifetime is much longer than that of charged particles and atomic oxygen and nitrogen species. The streamers also propagate in the volume between the cellulose particles. The cellulose surface in contact with a streamer is subjected to short-lived plasma species, which cause surface modification; the surface of the cellulose powder is thus modified. The modification includes the oxidation and nitridation of the very thin surface film and etching by forming volatile molecules such as CO, CO_2_, H_2_O, HCN, etc. [30,31,32,33,34]. The bulk cellulose is not affected by the influence of short-lived plasma particles. Thus, the high glucose yield reported by Benoit et al. [27] cannot be explained by such surface modifications. The long treatment time (hours) indicates other reactions, such as the diffusion of long-lived plasma species in the bulk cellulose or the propagation of the surface defects inside the bulk cellulose. The microcrystalline cellulose, however, is not known for such effects, so the rather large glucose yield, even in the absence of any catalyst, may be explained by the reactions triggered by plasma species that can penetrate the bulk cellulose. The only possible plasma species of larger penetration depth into organic matter are energetic photons. Plasma glows and, therefore, emits radiation in a broad range of photon energy from infrared (IR) to vacuum ultraviolet (VUV) [35,36]. If the energy of such photons is above the threshold needed for bond scission, the photons cause dangling bonds in the cellulose chain, which are occupied by water molecules. The net effect of irradiation with energetic photons is thus cellulose bond scission and formation of monomers (sugars), dimers, oligomers, and the like. Atmospheric-pressure plasma sustained in the air is not known as an extensive source of energetic photons [37], which may explain the long treatment times (hours) needed for significant glucose yields.

The same team also probed the combination of ball milling and plasma treatment [38]. Ball milling is among the standard methods for cellulose pre-treatment and is an alternative to chemical hydrothermal treatment with catalysts [39,40,41,42]. The combination of plasma treatment and ball milling resulted in the degree of polymerization down to 36. The cellulose was thus efficiently converted to low-molecular-weight oligomers. The microcrystalline cellulose (Avicel PH105) was first milled to obtain partial de-crystallization, as confirmed by X-ray diffraction (XRD). The milling did not influence the degree of polymerization, so the authors concluded that the milling only influenced the crystallinity. Then, the milled cellulose powder was placed in the same plasma reactor as reported in [27] and illustrated in Figure 2, and the discharge conditions were the same as in [27]. The plasma treatment caused significant solubility in dimethylsulfoxide (DMSO), which increased with the plasma treatment time and stabilized at about 80 wt.% (70 g/L) after treating the milled cellulose with gaseous plasma for an hour. This observation was explained by the extensive depolymerization of the amorphous phase upon plasma treatment. Even in pure water, the solubility was 37 wt.%. The authors also reported yellowing of the powder after prolonged plasma treatment, which was explained by the degradation of the oligomers. The pH of cellulose powder dispersed in water dropped to 2.5 (from the initial 5.6) after an hour of plasma treatment when the cellulose concentration of 15 g/L was used. The acidity of plasma-treated media is explained elsewhere [43,44]. Some experiments were performed using air plasma of different humidity and the authors found insignificant variations, so they concluded that the water needed for depolymerization arises from the powdered cellulose, not from the gas phase. The authors suggested the crucial role of nitric oxides in depolymerization. This suggestion was partially based on detecting the ammonium formate in the oligomers. The conclusion was that the plasma treatment ‘activated’ the water in the polysaccharides, which promotes the cleavage of the glycosidic bonds. The yield of glucose was determined after mixing with water and heating to temperatures between 100 and 150 °C for an hour. In the absence of an A35 catalyst, the glucose yield was 19 wt.% at 120 °C, and it was 48 wt.% when the A35 catalyst was added to the hot water. The ‘water activation’ refers to the dissociation of water inside the bulk cellulose due to the absorption of VUV photons.

Hilgert et al. [45] did not use a plasma treatment but reported a significant depolymerization of cellulose using a combination of mechanical, chemical, and hydrogen treatment. Hydrogen was introduced into the suspension at the pressure of 50 bar using a Ru/C catalyst, and the conversion rate of cellulose was practically 100% when the microcrystalline powder was impregnated with sulfuric acid and milled for 3 h. Up to about 80% of cellulose was transformed into 6-C sugars. Apparently, hydrogen adds to the efficiency of cellulose transformation into sugars.

Delaux et al. [46] also used plasma sustained by a DBD discharge at atmospheric pressure to depolymerize microcrystalline cellulose AVICEL 200. The degree of polymerization depended on the powder size and the cellulose crystallinity index and was between 150 and 200. The depolymerization rate increased with increasing treatment time during the first 10 min of plasma treatment and stabilized at about 40 (for a crystallinity index of 20) to 80 (for a crystallinity index of 80). A bipolar pulsed power supply at a maximum voltage of 10 kV and a frequency of 2.2 kHz was used to sustain the plasma in the DBD reactor with an electrode gap of 4 mm. The reactor was almost identical as that in Figure 2. The authors reported a discharge power of 15 W. The plasma treatment caused a dramatic increase in the saccharides’ solubility, confirming the cellulose powder’s chemical modification. The solubility increased with decreasing the cellulose crystallinity index. The powder with a crystallinity index below 10 µm treated with plasma for 15 min was almost completely soluble in DMSO, while 50% solubility was observed for the same powder in water. X-ray photoelectron spectroscopy (XPS) determined that the concentration of O–C=O bonds increased to 18 at.% after the plasma treatment, while it was at the edge of the XPS detection limit for untreated cellulose. Simultaneously, the concentration of the C–O bonds decreased from 73 to 50 at.%. These results were explained by the scission of bonds in the cellulose and the termination of many oligosaccharides and/or monosaccharides with –COOH functional groups on the powder surface. Any oxidation of the bulk cellulose was examined using various other techniques that are not as surface-sensitive as XPS, such as ^13^C cross-polarization and magic-angle-spinning nuclear magnetic resonance spectroscopy, Raman spectroscopy, gas chromatography coupled to mass spectrometry after methylation analysis, and matrix-assisted laser desorption/ionization time-of-flight mass spectrometry, and the authors found marginal (if any) oxidation of the bulk cellulose. The authors thus concluded that chemical modification, which is different from oxidation, occurred in bulk cellulose.

**Figure 3:**
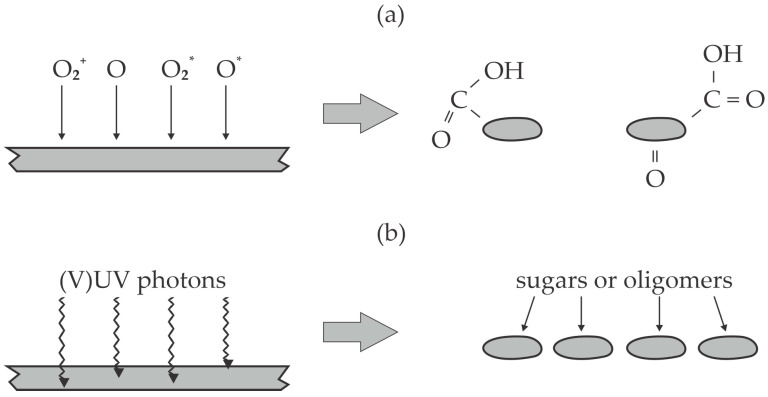
An illustration of the surface (**a**) and bulk (**b**) modification of cellulose upon treatment with air plasma sustained in the air; (**a**) the cellulose surface is exposed to reactive plasma species (molecular ions O_2_^+^, atoms in the ground state O, and molecules and atoms in excited states O_2_*, O*), which cause quick depolymerization and the formation of highly oxidized fragments; (**b**) the energetic photons penetrate the cellulose powder, where they cause depolymerization.

As already mentioned, the diffusion of oxygen inside bulk cellulose is improbable, so the results reported by Delaux et al. [46] can be explained by the bond scission in the bulk cellulose and, thus, depolymerization. The effect is illustrated in Figure 3. Figure 3a illustrates the reactions on the cellulose surface. The chemically reactive species—like positively charged oxygen ions, O atoms, and oxygen molecular and/or atomic metastables—cause partial oxidation of the cellulose chain. The effect is the formation of low molecular weight fragments terminated with carboxyl groups, as illustrated in Figure 3a. Figure 3b illustrates the reactions in the bulk cellulose (which is not achievable by chemically reactive plasma species). The energetic photons cause bond scission, the dangling bonds react with water molecules, and the net effect is depolymerization without significant oxidation. The penetration depth of energetic photons—with energy capable of bond scission in the polymer chains—in cellulose is yet to be determined, but the available results on other polymers indicate it should be between 100 nm and 100 µm and should increase with decreasing photon energy [47]. As already mentioned, the atmospheric-pressure plasma sustained by high-impedance discharges like DBD is not an extensive source of photons, so the limiting factor for cellulose depolymerization and increased solubility is the flux of energetic photons. Certainly, the plasma treatment gives the best results for thin cellulose deposits; in particular, energetic photons will never penetrate thick (macroscopic) cellulose samples.

The same team prepared a tutorial review of methods for the depolymerization of cellulose materials [48]. They reported that their plasma caused the cleavage of β-1,4 glycosidic bonds, followed by random re-polymerization. The net effect of plasma treatment was, thus, a formation of various glucans with preferential cleavage of α/β-1,6 glycosidic linkages. The re-polymerization of glycosyl radicals was explained through the C–O–C bond with the dominant formation of α/β-1,6 linkages, but the side oxidation was reported to be surface-limited. The plasma treatment was highly selective to the glycosylation reaction and caused no degradation of sugars, which was explained by the relatively low temperature of the cellulose powder during the plasma treatment. However, the authors also reported that the depolymerization did not occur at temperatures below 40 °C. Such a temperature is easily achieved upon the plasma treatment of all polymers because of the exothermic surface reactions, such as the neutralization of charged particles, the relaxation of metastables, and the heterogeneous surface recombination of oxygen atoms [49]. The authors [48] also mentioned that cellulases could not hydrolyze the glucans produced by plasma treatment of cellulose. However, when a solid acid catalyst (Amberlyst-35) was added to the water solution, depolymerization by hydrolysis yielded glucose as large as 60%.

**Figure 4:**
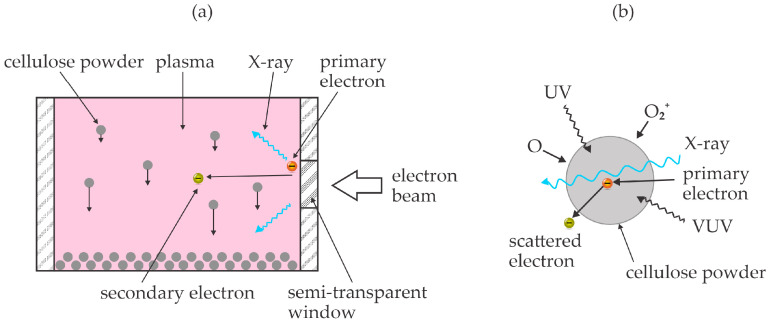
An illustration of the experimental setup (**a**) and penetration of energetic photons and electrons through cellulose powder (**b**). (**a**) Cellulose powder drops through plasma by gravitation and is subjected to plasma species, primary and secondary electrons, as well as X-rays; (**b**) reactive oxygen species cause surface oxidation, while UV, VUV, and X-ray radiation, as well as fast electrons, cause bond scission inside the cellulose powder.

Vasilieva et al. [50] used an electron-beam plasma to treat various polysaccharides and monitored the evolution of low-molecular-weight products. Unlike previous authors, they sustained homogeneous plasma in a rather large volume at a pressure of about 7 mbar. The working gas was pure oxygen. The cellulose powder was rather uniformly treated in the plasma chamber by rotation of the mixing device. The authors selected treatment times of 2 and 10 min. The chamber temperature was kept at about 40 °C during the plasma treatment. The treated powder was then used for suspensions either in pure water or water with an addition of 5% NaOH. The yield of soluble molecular fragments in water was about 2% for untreated powder and increased to 23 and 77% after treating the powder in oxygen plasma for 2 and 10 min, respectively. The yield was much larger in the alkaline liquid and was 100% for cellulose powder treated for 10 min. The polymerization degree dropped significantly as well. While the average polymerization degree was reported to be about 1000 for untreated powder, it dropped to about 150 for powder plasma-treated for 2 min and about 10 for powder plasma-treated for 10 min. Excellent treatment uniformity was ensured in the experimental setup used by Vasilieva et al. [50]. The authors attributed these excellent results to a combined effect of reactive oxygen species (Figure 3a), energetic photons (Figure 3b), energetic primary electrons (the energy is not explicitly stated but should be above 100 keV) and secondary electrons (energies up to about 1 keV are reported). The authors compared their technique with standard chemical hydrolysis and found it very beneficial for the production of low-molecular-weight products but less efficient for the production of sugars. The basic concept reported by Vasilieva et al. [50] is shown in Figure 4. Oxygen plasma treatment of cellulose powder also causes the production of nano-cellulosic fibrils from microfibrils, which was found beneficial for the dissolution of the oxidized hemicelluloses in water [51].

**Figure 5:**
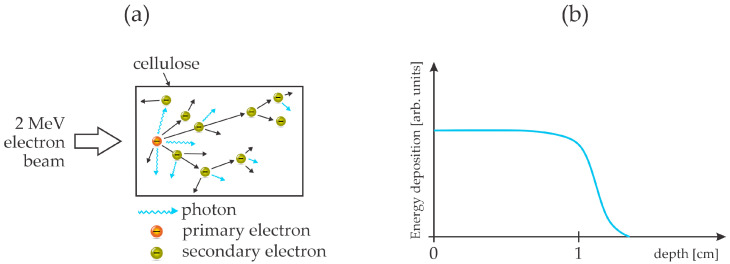
An illustration of the interaction of MeV energetic electrons with cellulose (**a**) and the distribution of the energy released inside the bulk cellulose (**b**).

The cellulose treatment with energetic electrons was also recently reported by Jang et al. [52]. Pre-hydrolyzed kraft birch pulp was ground by milling to obtain a fine powder. The lingo-cellulose powder was then exposed to an electron beam at the energy of 2 MeV. The penetration depth of such energetic electrons in organic matter is roughly 1 cm [53]. The chemical transformation of the cellulose upon irradiation with the energetic electrons was studied by various techniques. The electron dose was 1 or 2 MGy. As expected, the treatment caused the fragmentation of the cellulose chains down to oligomers. Some oxidation was also observed, but these reactions were found to be negligible compared to the depolymerization. Unfortunately, the authors did not report the glucose yield or a fraction of soluble matter before or after the treatment. The 2 MeV electrons are very energetic and cause radiation damage to any organic material. The scattering of such energetic particles triggers an avalanche of both secondary electrons and photons in a broad range up to gamma rays. Both secondary electrons and energetic photons break bonds in organic material, and the large energy of these species prevents selectivity. The effect of energetic electrons penetrating organic matter like cellulose is illustrated in Figure 5. The drawbacks of this method are the significant heating and limited availability of dispersed electron-beam sources of such large energy (MeV).

Some authors tried to explain the chemical reactions upon the treatment of polysaccharides with gaseous plasma species. The surface reactions upon the treatment of lignocellulose with air plasma containing reactive oxygen species were elaborated by Cao et al. [54]. They used density functional theory (DFT) to calculate the reaction pathways. They found that oxidative plasma species cleave the lignin C–C bonds and cause the formation of highly oxidized molecular fragments. They found the lowest decomposition energy of about 50 kcal/mol for the C_β_–O bond. The experiments were performed using plasma sustained in the air by a DBD discharge with a discharge power as large as 4.5 kW, and a plasma treatment time as low as 1.5 s. They performed XPS characterization of the treated samples and found the concentration of O–C=O bonds on plasma-treated samples to be about 11 at.%, close to the value reported by Delaux et al. [46]. The plasma treatment thus facilitated the rapid oxidation of the surface of the lignocellulose powder when powerful discharge was used.

Short plasma treatment times were also selected by Lusi et al. [55]. Plasma was sustained in the air at atmospheric pressure by a DBD discharge operating at a voltage of 15–20 kV. The goal of their study was the production of levoglucosan from cellulose. Cellulose powder was placed onto a dielectric plate, and plasma was sustained at the power of about 2 W. The plasma-treated cellulose powder was then pyrolyzed at the temperature of 450 °C. The levoglucosan yield upon pyrolysis was about 60 wt.% for untreated powder and increased to about 70 wt.% for samples treated with air plasma for 20 s at a discharge voltage of 15 and 17.5 kV. Further treatment with plasma caused minimal differences in the levoglucosan yield. Interestingly, the yield was much lower (up to about 65 wt.%) for cellulose samples treated with air plasma at a voltage of 20 kV. The cellulose treatment was also performed in argon plasma at the same discharge parameters, and the levoglucosan yield was somehow higher than for the same samples treated with air plasma. The solubility of the cellulose powder in DMSO was 43% for untreated powder and increased to 55% and 58% for cellulose treated with air and argon plasma for 30 s, respectively. The plasma-treated cellulose was barely soluble in water, but liquid chromatography–mass spectrometry detected some oligosaccharides. The crystallinity also dropped after plasma treatment, especially when using argon plasma. Argon is a noble gas, so it does not interact chemically with organic matter. Structural modifications observed after treating the cellulose powder with argon plasma should be attributed to bond scission triggered by the absorption of energetic photons, as illustrated in Figure 3b. Electron paramagnetic resonance showed a pronounced aging effect, which was explained by converting cellulose radicals to non-radical species, most likely by reacting with oxygen during storage for a few hours. The aging influenced the levoglucosan yield significantly. Namely, the yield became comparable to untreated samples already 12 h after the plasma treatment.

Lamine et al. [56] provided comprehensive theoretical results on the depolymerization of cellulose and similar materials when treating them with plasma species. The proposed major reaction pathway was the interaction of a hydroxyl radical with a hydrogen atom bonded to carbon on the cellulose chain, which causes the formation of a dangling bond and the spontaneous formation of a carboxyl group, which terminates the resultant oligosaccharide. The reaction is similar to that illustrated in Figure 3a, except that the reactive oxygen particle from plasma is not atomic oxygen but the OH radical. The abstraction of hydrogen by OH radicals has already been reported for polyolefins [57]. The reaction is highly probable because the OH radicals exhibit even a larger oxidation potential than O atoms.

### 2.3. Treatment of Water Suspension of Cellulose

**Figure 6:**
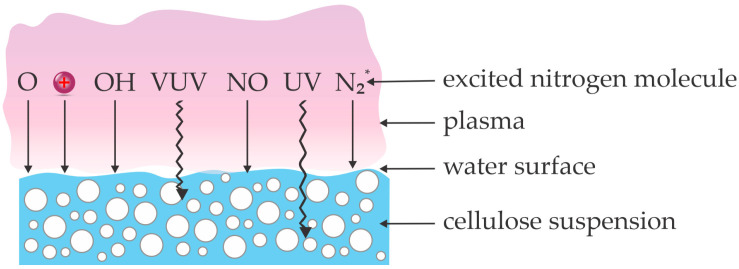
An illustration of interaction between plasma species and radiation with cellulose suspension.

An alternative to the treatment of dry powder is suspending cellulose powder in a liquid (typically water), and treating the suspension with gaseous plasma. Figure 6 depicts a concept of plasma treatment of water suspension used by the reviewed authors. Plasma is sustained in the gas phase above the liquid surface, with the gas above the water suspension being humid. The chemically reactive species like N_x_O_y_ molecules, OH, O, and N radicals, as well as positively charged ions, should dissolve in the liquid to cause any in-liquid chemistry, considering Henry’s coefficients for the solubility in water. The photons penetrate the water surface, and their penetration depth in pure water is large for UV photons and short for VUV photons [58,59]. The modification of cellulose suspended in the liquid is a consequence of the synergy between the photons and the dissolved chemically reactive species.

Grbic et al. [60] used corn stalks as a cellulose-containing raw material suspended in water and treated them with gaseous plasma sustained above water at atmospheric pressure in argon. A small plasma jet arising from the powered needle was used to treat the powdered corn stalks in a water suspension for 10–30 min. The cellulose-containing suspension was stirred during the plasma treatment to ensure a reasonably uniform treatment of 0.5 g of biomass. Iron salt was added after the plasma treatment, and the suspension was left at 34 °C for 24 h to allow for the Fenton reaction to progress. The final step in the sample preparation was rinsing with distilled water and diluted oxalic acid. The concentration of lignin was determined after each processing step. Half an hour of plasma treatment enabled a decrease in the lignin concentration by 19%. The authors attributed this to the synthesis of hydrogen peroxide in the suspension. Figure 6 indicates that the peroxide could form either by the interaction between two OH radicals or by the interaction of an oxygen atom with a water molecule. The suspension pH was about 3, and the lignin degradation in such an acidic environment was also supposed to be an effect of hydroperoxyl anion, hydroxyl radicals, and superoxide. Lignin degradation was up to 40% for samples treated first with plasma and then by Fenton reaction. The combined treatment also enhanced hydrolysis because the amount of hexose was over twice as much as for materials not treated with plasma, and the concentration of pentose was half of that obtained in a control experiment (enzymatic hydrolysis using a commercial mixture of cellulases). The results reported by Grbic et al. [60] could be explained as follows: An atmospheric-pressure plasma jet sustained in pure argon is an extensive source of VUV radiation arising from resonant atomic states and excimers [19]. The VUV radiation is already partially absorbed in the gas phase, but the remaining photons will be absorbed in the surface of the water suspension [20] by breaking H–OH bonds, and the final result will be the formation of hydrogen peroxide whose concentration saturates already at a rather low photon dose [59]. Detailed absorption spectra for VUV photons in water were provided recently by Bodi et al. [58]. The stirring of the suspension, as adopted by Grbic et al. [60], helps the uniform distribution of the highly oxidizing species in the liquid, and the species interact chemically with the lignocellulosic powder to cause partial depolymerization and lignin oxidation. The direct bond scission in lignocellulose by VUV photons should be minimal because the penetration depth of such radiation in water is below a micrometer [58]. In any case, the small size of the plasma jet and relatively costly argon limits the application of this technique for the depolymerization of cellulose.

Huang et al. [61] used plasma sustained by DBD discharge for the pre-treatment and hydrolysis of microcrystalline cellulose. The as-received cellulose exhibited a crystallinity of 83%. After prolonged milling, the crystallinity dropped to 57%. The microcrystalline cellulose was suspended in water. The interaction of gaseous plasma sustained in the air with water is illustrated in Figure 6 and explained in detail in the classic article by Bradu et al. [62]. Huang et al. [61] used a sinusoidal power supply operating at a voltage of 22 kV and a frequency of 15 kHz to power the discharge. Plasma was sustained in the air or moist argon, but differences in the cellulose depolymerization were negligible. Such a small difference leads to the conclusion that the type of reactive plasma particles does not influence depolymerization much. The plasma treatment times were up to 50 min. The plasma-treated water suspension of cellulose was then hydrolyzed in an acid solution at 180 °C, and the treatment time was 80 min. The degree of polymerization was found to decrease monotonously with increasing plasma treatment time. It was 215 for untreated samples and dropped to 105 after 50 min of plasma treatment. The experiments were also performed using milled cellulose, and the degree of polymerization dropped to 78. Huang et al. [61] attributed the depolymerization to the formation of OH, H_2_O_2_, and H_2_O^+^ in the water suspension, but the concentration of those species was not mentioned. The depolymerization effectiveness of microcrystalline cellulose was somehow inhibited by adding tertbutyl alcohol into the water suspension. The authors reported that the intramolecular and intermolecular O–H bonds in cellulose molecules were destroyed through reactions with the radical species. Furthermore, the authors explained that the β-1,4-glycosidic bonds between two anhydroglucose would also be cleaved by the constant attacking of the radical species. At optimal conditions, the total reducing sugar concentration increased by about 83% compared to cellulose not treated with gaseous plasma. It is worth mentioning that the dissociative absorption of energetic photons in water molecules limits the penetration of VUV radiation in pure water. According to Bodi et al. [58], pure water is practically transparent to UV radiation and soft VUV, but the absorption coefficient increases for six orders of magnitude (one million times) from the wavelength of 200 nm to 150 nm (photon energies from 6.2 to 8.3 eV).

Sakai et al. [63] treated a water suspension of cellulose in the early afterglow of atmospheric-pressure plasma sustained in argon with an admixture of 0.6 vol.% oxygen. Such an afterglow is supposed to be a rich source of neutral oxygen atoms in the ground state. The suspension of 60 mg of different cellulose samples in 3 mL distilled water was placed into a Petri dish and treated with the afterglow. The discharge parameters were not reported. The treated suspension was then subjected to enzymatic hydrolysis. The effect of pre-treatment with the afterglow was determined by measuring the concentration of reducing sugars, which increased monotonously with hydrolysis time. It slowly increased with increasing treatment time in the plasma afterglow. Untreated samples exhibited a sugar concentration of 0.7 mM, while it was about 1.5 mM for those treated for 20 min and after 1 h of enzymatic hydrolysis. The authors did not elaborate on the mechanisms involved in the pre-treatment of the cellulose, but they should be similar to those reported by Huang et al. [61].

Nastase et al. [64] treated inulin with a plasma sustained in the air, nitrogen, oxygen, or helium by the DBD discharge and reported depolymerization into fructo-oligosaccharides in 20 min. The degree of polymerization after such plasma treatments was below 5. The inulin was solubilized in ultrapure water, which was then treated with plasma at room temperature. The maximum fructose yield was about 7 g/L after treatment in the air plasma for 20–30 min and below 1 g/L after treatment at the same conditions with plasma sustained in other gases. An interesting experiment was performed by Cubas et al. [65]. They used argon plasma at atmospheric pressure to treat microalgae in water. The plasma was sustained by an alternating current discharge operating at a frequency as low as 60 Hz and a voltage of 17 kV. The current was limited to 30 mA, so the discharge power was about 500 W. An algae suspension was treated with plasma every second day for 3 min per day. The cellulose yield was doubled after 10 days of incubation. The pioneering work by Cubas et al. [65] indicates that plasma treatment is not only beneficial for the modification of polysaccharides but also stimulates their production.

## 3. Conclusions

The science of plasma depolymerization of cellulose is in its infancy, but some results show promise. The authors who measured the glucose yield reported a significant improvement due to plasma treatment, with up to about half of cellulose transformed into sugars. Furthermore, the concentration of water-soluble cellulose fragments increased dramatically even after rather short treatment times, thus making the cellulose depolymerization products suitable to be further degraded by enzymes. Still, lots of scientific work will have to be carried out before being able to evaluate the applicability of plasma treatment as a pre-step in biofuel synthesis. The advantage of plasma depolymerization over standard methods for pre-treatment of cellulose before enzymatic hydrolysis is in its ecological benignity and ability to transform water-insoluble cellulose to water-soluble oligosaccharides and sugars at neutral pH and room temperature. Still, a great deal of scientific work will have to be performed before plasma depolymerization can be used in experimental plants.

## 4. Roadmap

The most important scientific challenge is the evaluation of the effect of different plasma species on cellulose depolymerization. The literature survey and explanations provided in this review indicate the crucial role of species that are capable of penetrating cellulose, such as photons and fast electrons. Theoretical work would be welcome, but it should be supported by the measurements of the penetration depth of both photons and electrons versus their energy. The penetration depth of fast electrons should not depend much on the structure of organic matter, so a good approximation is offered by the results reported for other organic materials. Fast electrons cause an avalanche of less energetic electrons and photons in a broad range of energies, but the interaction of low-energy secondary electrons released inside the cellulose material is yet to be studied. The penetration depth of photons with energy just above the threshold for bond scission is unknown and should be determined experimentally, preferably by using well-defined beams of photons with adjustable energy, such as those available at (V)UV synchrotron beamlines.

Once these scientific results are obtained, technological challenges will become important. Knowing the estimated doses of plasma species, it is possible to perform simple calculations of economic justification. Currently, the largest cost in the production of second-generation biofuel is for enzymatic hydrolysis. Indeed, the plasma pre-treatment should cost much less than this step. While upscaling the plasma reactors does not represent a large technological challenge, it is questionable whether large reactors that operate at low discharge power density, often as low as kW/m^3^, will be capable of providing adequate fluxes of energetic plasma species.

## Data Availability

This is a review article; therefore, no new data were generated.

## Abbreviations

The following abbreviations are used in this manuscript:

VUV	Vacuum Ultraviolet
AC	Alternating Current
IR	Infrared
XRD	X-ray Diffraction
DMSO	Dimethylsulfoxide
UV	Ultraviolet
DBD	Dielectric Barrier Discharge
XPS	X-ray Photoelectron Spectroscopy
DFT	Density Functional Theory
